# The Gut Microbiome in Psychosis From Mice to Men: A Systematic Review of Preclinical and Clinical Studies

**DOI:** 10.3389/fpsyt.2020.00799

**Published:** 2020-08-11

**Authors:** Ann-Katrin Kraeuter, Riana Phillips, Zoltán Sarnyai

**Affiliations:** ^1^Laboratory of Psychiatric Neuroscience, Centre for Molecular Therapeutics, James Cook University, Townsville, QLD, Australia; ^2^Australian Institute of Tropical Health and Medicine, James Cook University, Townsville, QLD, Australia; ^3^Faculty of Health and Life Sciences, Psychology, Northumbria University, Newcastle upon Tyne, United Kingdom

**Keywords:** gut microbiota, schizophrenia, early life events, inflammation, microbiota metabolites, stress

## Abstract

The gut microbiome is rapidly becoming the focus of interest as a possible factor involved in the pathophysiology of neuropsychiatric disorders. Recent understanding of the pathophysiology of schizophrenia emphasizes the role of systemic components, including immune/inflammatory and metabolic processes, which are influenced by and interacting with the gut microbiome. Here we systematically review the current literature on the gut microbiome in schizophrenia-spectrum disorders and in their animal models. We found that the gut microbiome is altered in psychosis compared to healthy controls. Furthermore, we identified potential factors related to psychosis, which may contribute to the gut microbiome alterations. However, further research is needed to establish the disease-specificity and potential causal relationships between changes of the microbiome and disease pathophysiology. This can open up the possibility of. manipulating the gut microbiome for improved symptom control and for the development of novel therapeutic approaches in schizophrenia and related psychotic disorders.

## Introduction

The gut microbiome has recently received considerable interest, due to its potential role in maintaining health and in the pathophysiology of chronic diseases (1). Our understanding of the involvement of the gut microbiome in diseases is fast increasing due to the emergence of new molecular biological techniques (2). In healthy individuals, the gut microbiome, which consists of more than 100 trillion bacteria (3, 4), has a symbiotic relationship with enteric cells to influence physiological function (5). The gut microbiome is highly variable between healthy individuals (3), with twins only sharing 50% of their species-level bacterial taxa (6). This interindividual difference is shaped through host-extrinsic, host-intrinsic, and environmental factors (2, 3). Host-extrinsic factors include lifestyles, such as physical activity, cultural habits, medication, and diet (2). Environmental factors altering the microbiome include the local environment and maternal transmission (2). Host-intrinsic factors, which might shape the gut microbiota, are genetics, sex, innate, and adaptive immunity, as well as metabolic factors, i.e., body mass index (2, 7). Although interindividual differences exist in healthy individuals, studies have demonstrated clear separations of the gut microbiome in chronic diseases such as individuals with allergies (8–10), celiac disease (11), gastric cancer (12), inflammatory bowel disease (13, 14) including Crohn’s disease (15) and ulcerative colitis (3, 16), obesity (3, 17, 18), anorexia (17, 19), and type 2 diabetes mellitus (20) compared to healthy controls. The functional importance of the gut microbiome was demonstrated by the transfer of the gut microbiome from obese to germ-free mice resulting in obesity (21).

Bidirectional communication has been well established between the gut and the brain and its importance for maintaining neuronal, hormonal and immunological homeostasis has been recently demonstrated (22). A damage to the integrity of the gut-brain communication results in altered brain function and behavior (23). More recently, the importance of the gut-brain axis has been highlighted as a possible contributing factor, among many others, such as genes, early environment and nutrition, in the development of neuropsychiatric disorders (5, 22).

Schizophrenia is a heterogeneous, chronic neurodevelopmental psychiatric spectrum disorder influenced by a hitherto poorly understood interaction between genetic and environmental factors (24). It affects about 1 in 100 people (1%) worldwide (25). A variety of different pathophysiological mechanisms have been proposed, such as the dopamine hyperactivity in certain brain systems (26, 27), impaired glutamate neurotransmission (28), and a disruption of the brain glucose and energy metabolism (29–32). It has been conceptualized that multiple environmental “hits” on the background of a genetic predisposition are required for its development (33, 34). Genome-wide association studies have shown that schizophrenia is a polygenic disorder with a complex array of contributing risk loci across the allelic frequency spectrum (35, 36). Environmental events throughout development and adulthood, such as viruses before birth, method of delivery, birth complications, and psychosocial traumas, are important in the pathophysiology of schizophrenia (27) and the shaping of the gut microbiome (37–39). Most recently, a study demonstrated that mice receiving feces from individuals with schizophrenia showed a behavioral phenotype that is consistent with that have been seen in animal models of schizophrenia and depression (40). This finding demonstrates that a constituent of the fecal matter have effect of brain function and behavior of the host and strengthen the suggestion that the microbiome might contribute to behavioral symptoms in psychosis (41).

In this systematic review, we summarize the most recent findings on the gut microbiome in psychosis, including animal models and clinical data. Furthermore, we identified potential factors particular to psychosis, which may contribute to the altered gut microbiome. The methodology of the studies covered was not described in details as these were extensively reviewed elsewhere (42). Compared to previous reviews (43) we provide detailed discussion of factors such as antipsychotic use, lifestyle and environmental factors as well as the potential pathological role of the microbiome in psychosis relevant to microbial changes.

## Methods

### Eligibility and Inclusion Criteria

We included original articles investigating preclinical and clinical studies exploring the fecal microbiome in animal models of schizophrenia and individuals of all ages with psychosis or at high risk and schizophrenia along with respective controls. Only studies published in English were included without a date restriction throughout the database search.

### Database Search Strategy

This study followed the Preferred Items for Reporting Systematic Reviews and Meta-analysis Protocols (PRISM-P) (44). One author (AKK) conducted a Scopus, Web of Science, and PubMed database searches until the cut-off date of 14/02/2019. In all databases, free-text terms included (microbiota OR gastrointestinal microbiome OR microbiome OR microbio*) AND (schizophreni* OR “Dementia Praecox” OR psychotic OR schizoaffective OR psychoses OR psychosis). The search was limited to original articles, and therefore we excluded reviews, meta-analyses, and systematic reviews. The reference lists of eligible papers were manually screened for further relevant articles.

### Report Selection

One of the authors (A-KK) determined the eligibility of papers by screening titles and abstracts for relevance. Eligible documents were then read as a whole to analyze if the articles matched the inclusion criteria. Excluded articles were documented, and reasons were given for exclusion.

### Data Extraction

A-KK extracted information from relevant publications such as animal and patient characteristics. Study characteristics for animal experiments included strain, sex, number, age, and weight of animals and “schizophrenia” induction method, length of study, the timing of fecal sample collection, microbiota, and other findings of the study. Human studies were characterized by the number of participants, gender, age, exclusion and inclusion criteria, microbiota findings, and other findings within the study.

### SYRCLE’s Risk of Bias Analysis

The overall risk of bias (RoB) was assessed in animal studies using an adapted SYRCLE’s risk of bias tool (45). All ten entries of the SYRCLE’s RoB tool were assessed by the authors (A-KK, RP) relating to selection, performance, detection, attrition, reporting, and other biases (45). All individual entries were assigned as “low RoB,” “high RoB,” “unclear,” or “not feasible.” A parameter was determined “unclear” if the item was not mentioned in the publication. The only exception to this was item 8, where ‘not mentioned’ was scored “high RoB.” For one article, it was not feasible to assess housing conditions (items 3, 4, and 5) due to the nature of that particular animal model of schizophrenia (46). Furthermore, studies were assessed for quality by answering the categories: (1) conflict of interest stated, (2) power analysis or sample size calculation, (3) experiment blinding at any level, and (4) randomization at any level.

### STROBE Risk of Bias Analysis

Human studies were assessed using adapted STROBE assessment criteria, including 32 subsections, which were scored for all six studies. A-KK and RP assessed the completeness of reporting (CoR) score (CoR (%)=(yes/(yes+no))*100) by answering each recommendation in the STROBE statement with “yes” or “no.”

### Microbiome Methodological Consideration

All studies were investigate for their CoR for microbiome relevant methodology. Categories range from sample preparation, handling to analysis of samples. The CoR score was calculated as described in the previous section. The overall CoR score was calculated for all studies, animal studies alone and human studies alone.

## Results

### Database Search

**Figure 1:**
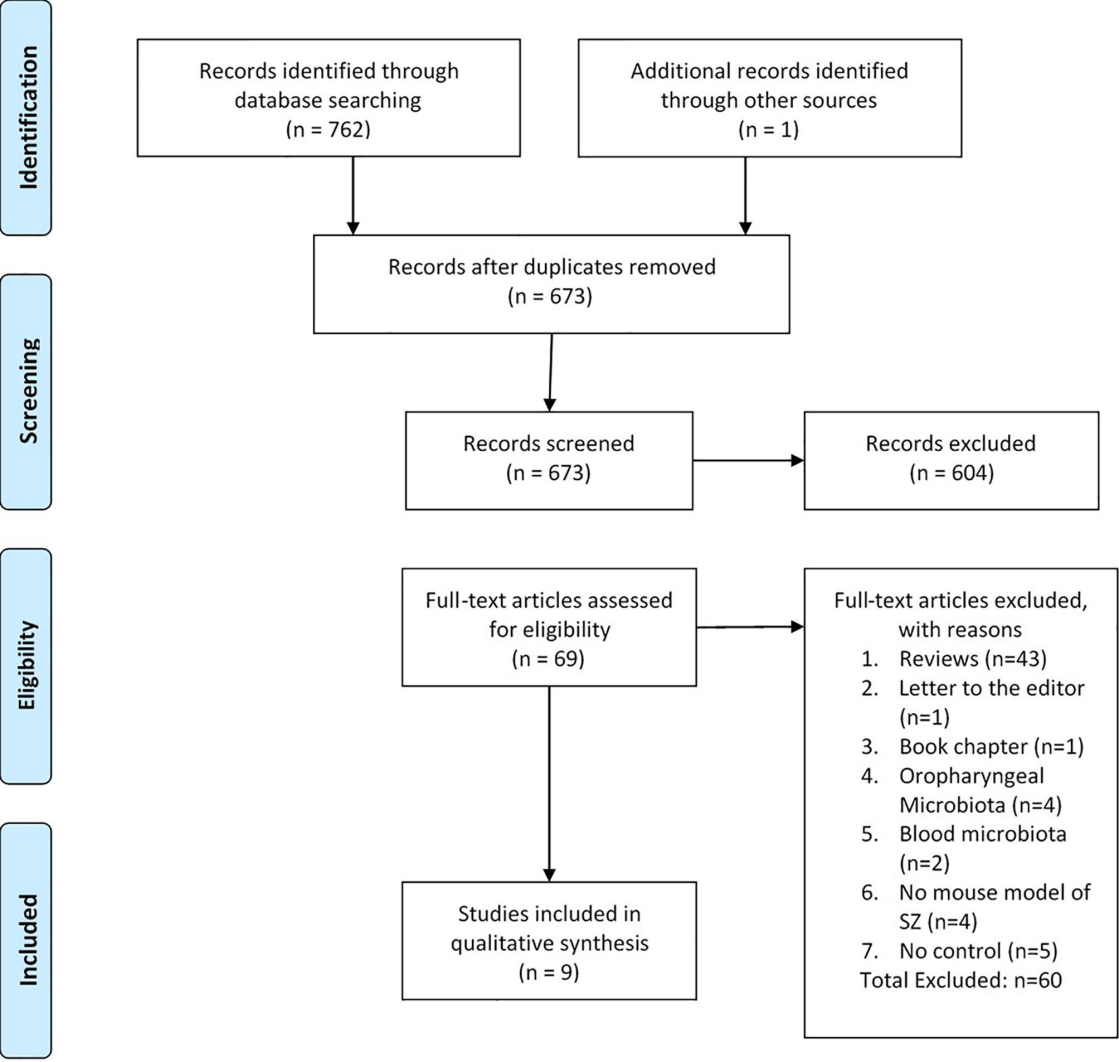
Flow diagram (47).

The initial search yielded 763 documents, including one additional record identified through other sources. After exclusion of duplicates, 673 articles remained and were included for evaluation of titles or abstracts, which resulted in 69 records for full-text article review. During full-text article review, 60 articles were excluded because they were not original research articles (reviews, n=43; letter to the editor, n=1; book chapter, n=1), referred to microbiomes other than the gut microbiome (oropharyngeal, n=4; blood, n=2), was not a mouse model of schizophrenia (n=4), or reported no control (n=5). One additional article was found during scanning of the references. This search resulted in nine articles included in this systematic review (**Figure 1**).

### Study Characteristics

#### Animals

Preclinical publications included within this review used three different types of translationally validated animal models. A neurodevelopmental model, the maternal immune activation (MIA), which takes advantage of the finding that maternal infection during pregnancy increases the risk of developing schizophrenia in the offspring, by injecting pregnant females with viral mimic polyriboinosinic–polyribocytidilic acid (Poly I:C), which disrupts prenatal and early postnatal development (48). A pharmacological model, the N-methyl-D-aspartate (NMDA) receptor hypofunction model induced by administration of the NMDA receptor antagonist phencyclidine, is based on the findings that the administration of NMDA antagonists [phencyclidine (PCP) and ketamine] induce schizophrenia-like behaviors (49, 50). Social isolation is a major “psychological” stressor that has been used over the years to induce a behavioral and neurochemical phenotype corresponding to schizophrenia (51). This alteration has long-lasting effects on the brain and behavior (48). Articles describing preclinical results included 144 male Lister-Hooded rats investigated the microbiome in a pharmacological, phencyclidine (52), and a developmental model (social isolation) (46) (**Table 1**). In the neurodevelopmental model, the gut microbiome was assessed in ten C57/Bl6 offspring in the MIA group and ten respective controls with unknown sex (53). The two studies using rats had similar size rats of 100-130 g (46, 52); only one study reported that rats were 24 days old (46). C57BL/6N mice were six weeks of age (53). All studies analyzed microbiome using 16S rRNA gene MiSeq-based high throughput sequencing (46, 52, 53). All studies were supported by various sources of funding (46, 52, 53).

**Table 1:** Gut microbiota changes in animal models of schizophrenia.

	Strain, Sex (n), Age (weight)	Disease induction and length of study	Fecal sample collection timing	Microbiota changes	Other findings	Comments
Pyndt Jørgensen, Krych (52)	EXP1-4: Lister-Hooded rats, M (24), NA (100-130g)	EXP1-4: SZ: 5 mg/kg free base PCP i.p. Control: vehicle i.p. EXP4: addition ampicillin through the drinking water Length of study EXP1: 7 days PCP and 7 day washout EXP2: 7 days PCP and 28 day washout EXP3: 7 days PCP and 49 day washout EXP4: 7 days PCP and 7 day washout	EXP1: Day 16 EXP2: Day 37 EXP3: Day 58 EXP4: Caecum: Day 19	EXP1: No difference • α-diversity Altered • β-diversity Increased • *Roseburia (Phylum: Firmicutes)* • *Odoribacter* (Phylum: *Bacteroidetes*) Increased Locomotor activity positively correlated with: • *Lachnospiraceae* (Phylum: *Firmicutes*) • *Clostridiaceae* (Phylum: *Firmicutes*) • *Roseburia* (Phylum: *Firmicutes*) • *Clostridium* (Phylum: *Firmicutes*) • *Odoribacter* (Phylum: *Bacteroidetes*) EXP2: No difference • α-diversity • β-diversity Increased • unclassified genus bellowing to the S24-7 family (Phylum: *Bacteroidetes*) • Dorea (Phylum: *Firmicutes*)	EXP1-3: Increased • Locomotion EXP1 and 2: Decreased • NOR performance EXP3: No Change • NOR performance EXP4: Improved • Reversed cognitive deficits in No change • Locomotion	EXP1 and 4: • NOR on day 15 and16 • Locomotor activity on day 18 EXP2: • NOR on day 36 and 37 • Locomotor activity on day 39 EXP3: • NOR on day 57 and 58 • Locomotor activity on day 60
Dunphy-Doherty, O’Mahony (46)	Lister-Hooded rats, M (24), 24 days (100-130g)	Social isolation Control: group housed Length of study 63 days	Caecum: Day 86/87	No difference • α-diversity • β-diversity Increased: • Phylum: Actinobacteria • *Rhodococcus* (Phylum: *Actinobacteria*) • *Negativicutes* (Phylum: *Firmicutes*) • *Corynebacteriales* (Phylum: *Actinobacteria*) • *Bacillales* (Phylum: *Firmicutes*) • *Selenomonadales* (Phylum: *Firmicutes*) • *Nocardiaceae* (Phylum: *Actinobacteria*) • *Bacillaceae* (Phylum: *Firmicutes*) • *Veillonellaceae* (Phylum: *Firmicutes*) • *Prevotellaceae UCG-001* (Phylum: *Bacteroidetes*) • *Bacillus* (Phylum: *Firmicutes*) • *Defluvitaleaceae UCG-011* (Phylum: *Firmicutes*) • *Eubacterium oxidoreducens group* (Phylum: *Firmicutes*) • *Marvinbryantia* (Phylum: *Firmicutes*) • *Veillonella* (Phylum: *Firmicutes*) Decreased • *Clostridia (Phylum: Firmicutes)* • *Clostridiales (Phylum: Firmicutes)* • *Clostridiacae group 1 (Phylum: Firmicutes)* • *Peptostreptococcaceae (Phylum: Firmicutes)* • *Lachnospiraceae UCG-009* (Phylum: *Firmicutes*) • *Ocillospira* (Phylum: *Firmicutes*) • *Papillibacter* (Phylum: *Firmicutes*) *Correlations described in text	Increased • Locomotion (OF) Decreased • Defecation (OF) • Freezing first, 24,48 shocks (CFR) • Cells in dentate gyrus dual labeled for BrdU and NeuN • Il-6 and IL-10 in hippocampus No Change • Rearing (OF) • Grooming (OF) • NOR • EPM • Second shock (CFR) • Corticosterone (restrained) • IL-1b or TNF-α	• 56 rats received 5-bromo-20-deoxyuridine • Open field (day59) • Locomotor activity noval area (day 65) • NOR (day 66) • EPM (day73) • Conditioned Freezing Response (day79/80/81) • Restrained Stress and Sample collection (day 86/87)
Hsiao, McBride (53)	C57BL/6N offspring, Sex:unknown, Microbiota: n=10/group, Behavior: 16-75/group), 6 weeks at behavioral testing	Pregnant C57BL/6N mice were injected i.p. on E12.5 with saline or 20 mg/kg poly(I:C) Length of study 9 weeks	Unknown	No difference • α-diversity Altered • β-diversity Increased • *unclassified Bacteriodales* (Phylum: *Bacteroidetes*) • Porphyromonadaceae (Phylum: *Bacteroidetes*) • *Prevotellaceae* (Phylum: *Bacteroidetes*) • *Lachnospiraceae* (Phylum: *Firmicutes*) Decreased • *Ruminococcaceae* (Phylum: *Firmicutes*) • *Erysipelotrichaceae* (Phylum: *Firmicutes*) • *Alcaligenaceae* (Phylum: *Proteobacteria*) No change • *Clostrodia (Phylum: Firmicutes)* • *Bacteroidia (Phylum: Bacteroidetes)*	Increased • Intestinal permeability (3 week old offspring) • Gene expression (CLDN15) • IL-6 mRNA and protein • Repetitive behavior (marbles buried) Decreased • Gene expression (TJP1, TJP2, OCLN, and CLDN8) • IL-12p40/p70 • MIP-1a • Centre entries and time spent (OF) • PPI • Communication (ultrasonic vocalization) • Sociability (3CST) • Social preference (3CST)	Normalization with probiotic

SZ, schizophrenia; PCP, phencyclidine; NOR, novel object recognition; OF, open field test; CRF, conditioned freezing task; CST, chronic social defeat.

#### Human

Six eligible studies were identified, which investigated high-risk and ultrahigh-risk (UHR) individuals (4), first-episode psychosis (54), first-episode schizophrenia (55), and individuals with chronic schizophrenia (40, 56, 57) all compared to healthy controls (**Table 2**). High-risk (or at risk) state is the clinical presentation of those considered at risk of developing psychosis or schizophrenia. Such states were formerly considered as prodromes, emerging symptoms of psychosis, but this view is no longer maintained as a prodromal period can not be confirmed unless the emergence of the condition has occurred. Individuals are considered UHR for psychosis if they meet a set of standardized criteria including presumed genetic vulnerability (Trait), or a recent history of Attenuated Psychotic Symptoms (APS) or Brief Limited Intermittent Psychotic Symptoms (BLIPS) Yung, McGorry (58, 59). First-episode and chronic schizophrenia are defined in **Table 2**. A total of 321 patients and 273 healthy controls were investigated. All studies reported no significant differences between the experimental and control group for age, sex, and weight. Age varied widely between studies due to different stages of the disorder from 20.47 ± 4.57 to 54.7 ± 10.7. Common exclusion criteria included factors potentially influencing the gut microbiome such as gastrointestinal and endocrine disorders, previous antibiotic or probiotic treatment, alcohol and substance abuse. Inclusion criteria varied greatly between studies due to different baseline diagnostic criteria.

**Table 2:** Gut microbiota changes in schizophrenic patients and at risk individuals.

	Patient Characteristics(N, Gender, Age)	Exclusion/Inclusion criteria	Microbiota analysis	Microbiota findings at Baseline compared to control	Other findings	Comments
He, Kosciolek (4)	• HR (41M, 40F, 21.67 ± 5.75) • UHR (15M, 4F, 20.47 ± 4.57) • HC (37M, 32F, 23.13 ± 3.89)	Exclusion: • Gastrointestinal and endocrine diseases • Diagnosis with psychotic disorder and corresponding treatments • Last 3 month: alcohol, antibiotics, probiotics or any other oral or injectable medications	• One measure at baseline	No difference • α-diversity Altered: • β-diversity in HR and UHR Increased in UHR: • *Clostridiales* (Phylum: *Firmicutes*) • *Lactobacillales* (Phylum: *Firmicutes*) • *Bacteroidales* (Phylum: *Bacteroidetes*) • *Lactobacillus* (Phylum: *Firmicutes*) • *Prevotella* (Phylum: *Bacteroidetes*) • *Lactobacillus ruminis* (Phylum: *Firmicutes*) No Change • HR group	No difference: • Age and gender Increased: • Symptoms in UHR • Choline levels in UHR	• HC: no family history of mental illness • 37 HC did not agree with ^1^H-MRS • ^1^H-MRS: 7 HR and 2 UHR excluded
Schwarz, Maukonen (54)	• FEP (16M, 12, 25.9 ± 5.5) • HC (8M, 8F, 27.8 ± 6.0)	Exclusion: • Substance-induced psychosis and psychotic disorders due to general medical conditions Inclusion: • Score of at least 4 in the item assessing delusion (Usual Thought Content) and hallucinations (Brief Psychiatric rating scale)	• FEP: Morning of interview • HC: Sample at home and delivered them within a few hours to the laboratory • Baseline, 2 and 12 month	No difference • α-diversity Increased: • *Phylum Actinobacteria* • *Rhizobiales* (Phylum: *Proteobacteria*) • Bacillales (Phylum: *Firmicutes*) • *Lactobacillaceae* (Phylum: *Firmicutes)* • *Halothiobacillaceae* (Phylum: *Proteobacteria)* • *Brucellaceae* (Phylum: *Proteobacteria)* • unclassified *Micrococcineae* (Phylum: *Actinobacteria*) • *Lactobacillus* (Phylum: *Firmicutes)* • *Tropheryma* (Phylum: *Actinobacteria)* • *Halothiobacillus* (Phylum: *Proteobacteria)* • *Saccharophagus* (Phylum: *Proteobacteria)* • *Ochrobactrum*` (Phylum: *Proteobacteria)* • *Deferribacter* (Phylum: “*Deferribacteres”)* • *Halorubrum* (Phylum: *Euryarchaeota)* • Lactobacillus aciddophilus (Phylum: *Firmicutes*) • Lactobacillus grasser (Phylum: *Firmicutes*) • Lactobacillus saliva (Phylum: *Firmicutes*) • Lactobacillus reuter (Phylum: *Firmicutes*) • Lactobacillus fermen (Phylum: *Firmicutes*) • Desulfosporosinus acidphilus (Phylum: *Firmicutes*) • Bifidobacterium dentium (Phylum: *Actinobacteria*) • Tropheryma whipplei (Phylum: *Actinobacteria*) • Ochrobactum anthropi (Phylum: *Proteobacteria*) • Bartonella clarridgeiae (Phylum: *Proteobacteria*) • Franisella hispaniensis (Phylum: *Proteobacteria*) • Nitrosocococcus halophilus (Phylum: *Proteobacteria*) • Brucella canis (Phylum: *Proteobacteria*) • Saccharophagus degradans (Phylum: *Proteobacteria*) • Halothiobacillus neapolitanus (Phylum: *Proteobacteria*) • Deferribacter desulfuricans (Phylum: “*Deferribacteres”*) Decreased: • *Negativicutes* (Phylum: *Firmicutes*) • *Selenomondales* (Phylum: *Firmicutes*) • *Veillonellaceae* (Phylum: *Firmicutes)* • *Anabaena* (Phylum: *Cyanobacteria*) • *Nitrosospira* (Phylum: *Proteobacteria)* • *Gallionella* (Phylum: *Proteobacteria)* • *Thermococcos gammatolerans* (Phylum: *Euryarchaeota*) • *Leuconostoc gasicomitatum* (Phylum: *Firmicutes*) • *Nitrosomonas* spp. (Phylum: *Proteobacteria*) • *Anabaena variabilities* (Phylum: *Cyanobacteria*) • *Gallionella capsiferriformans* (Phylum: *Proteobacteria*) • *Chlorobium chlorochromate* (Phylum: Chlorobi) • *Nitrosospira multiformis* (Phylum: *Proteobacteria*) • *Xenorhabdus nematophila* (Phylum: *Proteobacteria*) In active SZ patients Increased: • *Lactobacillaceae* (Phylum: *Firmicutes)* Decreased: • *Veillonellaceae* (Phylum: Firmicutes *Correlations described in text	No difference: • Age, gender and several metabolic parameters (BMI, cholesterol, high and low density lipoproteins, glucose, insulin and triglycerides) • SZ patient less active Cofounders-no association: • Physical activity • Type of psychosis • Duration of antipsychotic treatment • Distribution of risperidone, quetiapine or olanzapine treatment • Intake of different food types over the week prior to sample collection	• Food habits and physical activity assessed • Fecal sample not collected if: antibiotic use during the past 3 months, chronic gastrointestinal disease, gastrointestinal surgery, or diagnosed celiac disease. • Microbiota clustering at intake was significantly associated with remission at follow-up
Shen, Xu (57)	• SZ (M36, F28, 42 ± 11) • HC (M35, F18, 39 ± 14)	Exclusion: • Last 3 month: Disease that may affect the stability of gut microbiota • Last 6 months: antibiotics, glucocorticoids, cytokines, large doses of probiotics and biological agents • Gastroscopy, colonoscopy or gastrointestinal barium meal • Last 5 years: major gastrointestinal tract surgery • Activity limitation • Changes in dietary habits • Alcohol abuse or dependence Inclusion: • SZ patients were diagnosed according to ICD-10 and received antipsychotic treatment in hospital or outpatient clinic • Illness duration ≤10 years and received antipsychotic drugs treatment **>** 6months; psychiatric symptoms were steady >3 months, and the PANSS evaluated the rate of change ≤20% and the total score of PANSS **≤**60.	• One measure at baseline	No difference • α-diversity Altered: • β-diversity Increased: • Phylum *Proteobacteria* • Phylum *Fusobacteria* • *Gammaproteobacteria* (Phylum: *Proteobacteria*) • *Fusobacteriia* (Phylum: *Fusobacteria*) • Enterobacteriales (Phylum: *Proteobacteria*) • Fusobacteriales (Phylum: *Fusobacteria*) • *Aeromonadales* (Phylum: *Proteobacteria*) • *Prevotellaceae (Phylum: Bacteroidetes)* • *Enterobacteriaceae (Phylum: Proteobacteria)* • *Succinivibrionaceae (Phylum: Proteobacteria)* • *Fusobacteriaceae (Phylum: Fusobacteria)* • *Veillonellaceae (Phylum: Firmicutes)* • *Lactobacillaceae (Phylum: Firmicutes)* • *Succinivibrio (Phylum: Proteobacteria)* • *Megasphaera (Phylum: Firmicutes)* • *Acidaminococcus (Phylum: Firmicutes)* • *Collinsella (Phylum: Actinobacteria)* • *Clostridium (Phylum: Firmicutes)* • *Klebsiella (Phylum: Proteobacteria)* • *Citrobacter (Phylum: Proteobacteria)* • *Methanobrevibacter (Phylum: Euryarchaeota)* • *Fusobacterium (Phylum: Fusobacteria)* • *Lactobacillus (Phylum: Firmicutes)* • *Phascolarctobacterium (Phylum: Firmicutes)* • *Desulfovibrio (Phylum: Firmicutes)* • *Collinsella aerofaciens (Phylum: Actinobacteria)* • *Bifidobacterium adolescentis (Phylum: Actinobacteria)* • *Prevotella stercorea (Phylum: Bacteroidetes)* • *Bacteroides fragilis (Phylum: Bacteroidetes)* • *Lactobacillus mucosae (Phylum: Firmicutes)* • *Lactobacillus ruminis (Phylum: Firmicutes)* Decreased: • Phylum *Firmicutes* • *Clostridia* (Phylum: *Firmicutes*) • *Clostridiales (Phylum: Firmicutes)* • *Streptococcaceae (Phylum: Firmicutes)* • *Alcaligenaceae (Phylum: Proteobacteria)* • *Lachnospiraceae (Phylum: Firmicutes)* • *Streptococcus (Phylum: Firmicutes)* • *Blautia (Phylum: Firmicutes)* • *Coprococcus (Phylum: Firmicutes)* • *Roseburia (Phylum: Firmicutes)* • *Roseburia faecis (Phylum: Firmicutes)* • *Blautia producta (Phylum: Firmicutes)* • *Collinsella plebeius (Phylum: Actinobacteria)* • Bacteroides eggerthii (Phylum: Bacteroidetes) *Correlations described in text	No difference: • Age, BMI, sex ratio, tobacco used and alcohol intake	
Yuan, Zhang (55)	• FES (M23, F18, 23.1 ± 8.0) • HC (M20, F21, 24.7 ± 6.7)	Exclusion • Autoimmune diseases, heart diseases, hepatobiliary and gastrointestinal diseases, blood diseases, diabetes neurological diseases, or psychiatric diseases other than FES • Pregnant or lactating women • A history of using any antibiotic or anti-inflammatory agent, or probiotic in the past month • A significant change in the living environment or diet in the past month • Significant diarrhea or constipation in the past month. *Healthy controls had the same exclusion criteria as patients; in addition, they had no previous history of any psychiatric diseases*. Inclusion • FES based on the DSM-IV criteria • Never been on antipsychotic medication • PANSS total score **N**60 points • Born through normal vaginal delivery • Normal body weight (BMI: 18.5**–**23.0).	• Baseline, 6,12,24 weeks of risperidone treatment	Increased: • Clostridium coccoides group (Phylum: *Firmicutes)* Decreased: • Bifidobacterium spp. (Phylum: *Actinobacteria)* • Escherichia coli (Phylum: *Proteobacteria)* • Lactobacillus spp. (Phylum: *Firmicutes)* No Change: • Bacteroides spp. (Phylum: Bacteroidetes*)* After 24 weeks risperidone- Increased: • Bifidobacterium spp. (Phylum: *Actinobacteria)* • Escherichia coli (Phylum: *Proteobacteria)* Decreased: • Clostridium coccoides group (Phylum: *Firmicutes)* • Lactobacillus spp. (Phylum: *Firmicutes)* No Change: • Bacteroides spp. (Phylum: Bacteroidetes) *Correlations described in text	After 24 weeks risperidone- Increased: • Weight • BMI • Fasting serum levels of glucose • Triglycerides • LDL • HOMA-IR • Serum levels of hs-CRP • SOD	
Nguyen, Kosciolek (56)	• SZ or schizoaffective disorder (14M, 11F, 52.9 ± 11.2) • HC (15M, 10F, 54.7 ± 10.7)	Exclusion • Other current major DSM-IV-TR Axis I diagnoses • Alcohol or other substance (other than tobacco) (within 3 months prior to enrollment) • Diagnosis of dementia • Intellectual disability disorder, • Major neurological disorder • Any medical disability that interfered with a subject’s ability to complete study procedures	• One measure • Home stool collection kits (samples returned *via* mail)	No difference • α-diversity Altered: • β-diversity Increased: • *Anaerococcus* (Phylum: *Firmicutes*) Decreased: • Phylum *Proteobacteria* • *Haemophilus* (Phylum: *Proteobacteria)* • *Sutterella* (Phylum: *Proteobacteria)* • *Clostrodium* (Phylum: *Firmicutes*) *Correlations described in text	No difference: • Age, gender, race Increased: • BMI (however, no differences in BMI classifications) • Psychiatric symptoms • Depression levels • Anxiety levels • Smoking • Medical comorbidity (diabetes and hypertension) Decreased: • Physical well-being	• 21 patients on antipsychotics at study onset
Zheng, Zeng (40)	• SZ (63) • HC (69)	Exclusion • Physical or other mental disorders • Illicit drug use • Antibiotics/probiotics within 1 month of study	•	Decreased: • α-diversity Altered: • β-diversity Increased: • *Veillonellaceae* (Phylum: *Firmicutes*) • *Prevotellaceae* (Phylum: *Bacteroidetes*) • *Bacteroidaceae* (Phylum: *Bacteroidetes*) • *Coriobacteriaceae* (Phylum: *Actinobacteria*) Decreased: • Lachnospiraceae (Phylum: *Firmicutes*) • Ruminococcaceae (Phylum: *Firmicutes*) • Enterobacteriaceae (Phylum: Proteobacteria) *Correlations described in text and comparison to depressive disorder and FMT from human to mouse	No difference: • Age, gender, BMI Increased: • Serum glutamine • Hippocampal GABA Decreased: • Stool and hippocampal glutamate	SZ were treated with a single antipsychotic drug: • Clozapine (n = 15) • Risperidone (n = 14) • Olanzapine (n = 9) • Chlorpromazine (n = 5) • Aripiprazole (n = 3) • Quetiapine (n = 3) • Remaining patients were treated with two of the above drugs in combination (n = 9) • Unmedicated (n = 5).

SZ, schizophrenia; HR, high-risk; UHR, ultrahigh-risk, HC, healthy controls; FEP, first-episode psychosis; FES, first-episode schizophrenia; BMI, body mass index; PANSS, Positive and Negative Syndrome Scale.

### Risk of Bias

#### Animals

**Figure 2:**
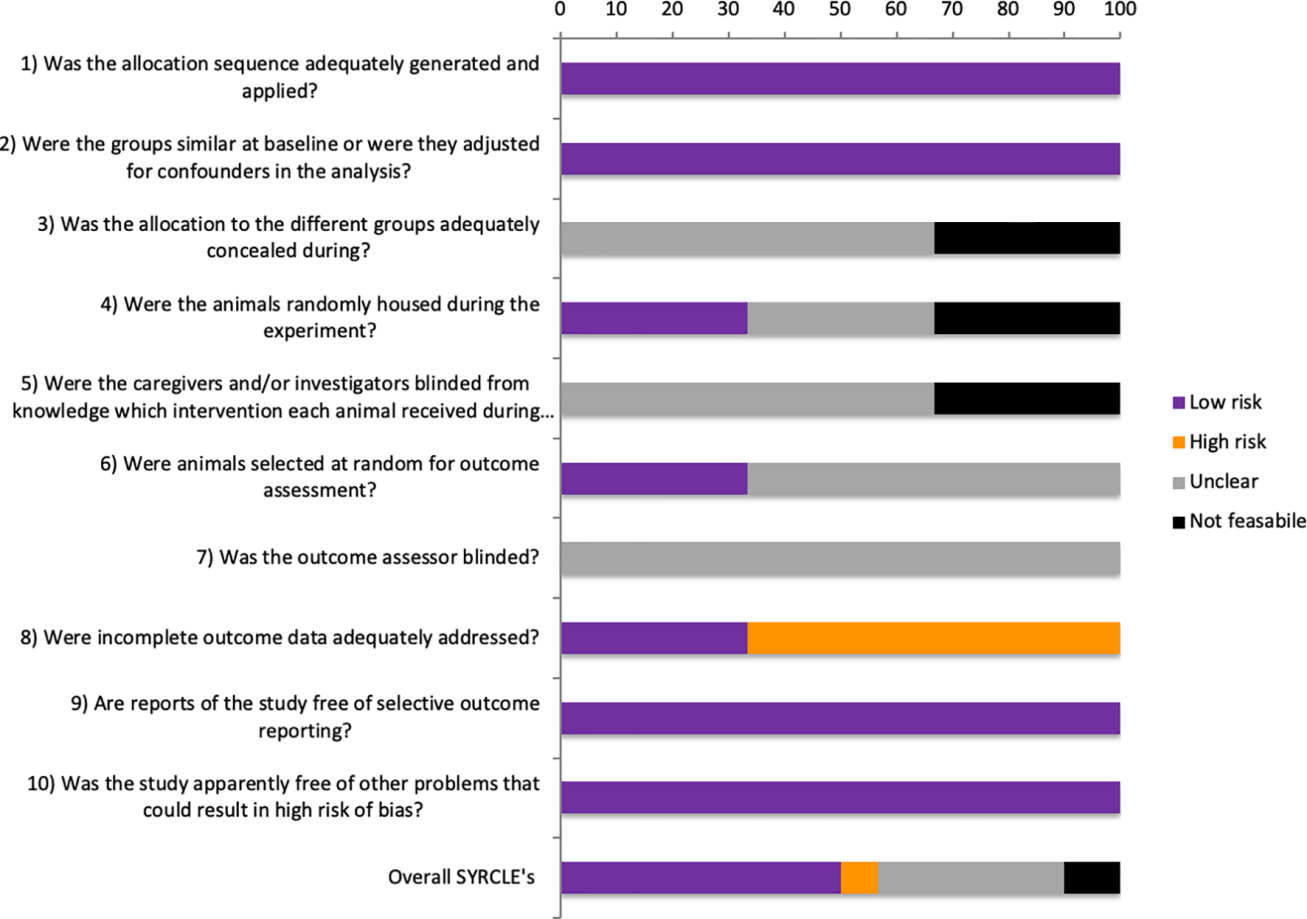
SYRCLE’s risk of bias tool.

Of the 30 SYRCLE entries of all three studies, 15 (50%) were low RoB, 2 (6.6%) were high RoB, 10 (33.3%) were unclear, and 3 (10%) were considered not feasible (**Figure 2**).

#### Quality Assessment

**Figure 3:**
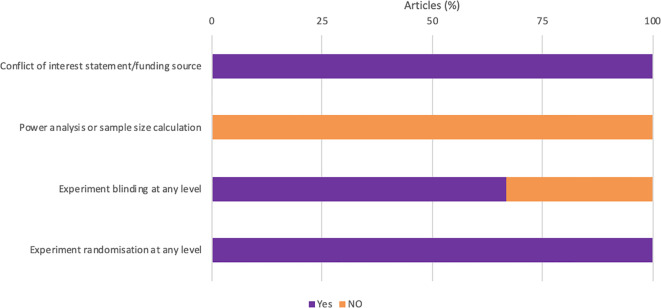
Quality assessment of all animal studies.

All studies were randomized and acknowledged their funding source (**Figure 3**). However, none of the studies reported how the sample size was estimated during the design of the experiment (**Figure 3**). One study failed to report if the experimenter was blinded at any level.

#### Human Studies

**Figure 4:**
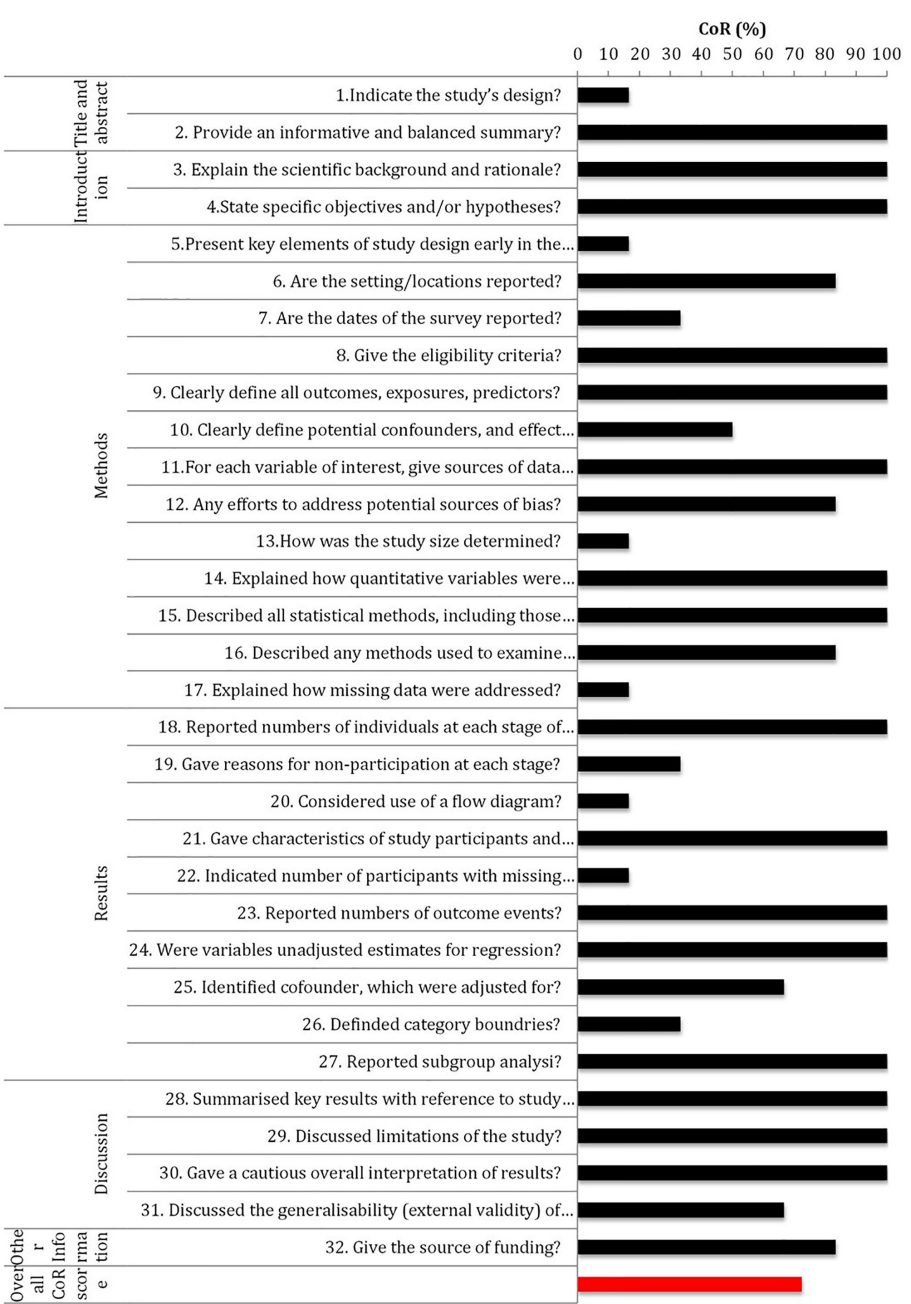
STROBE completeness of reporting analysis of human studies.

The analysis included a total of 192 entries (32 STROBE entries per study). Of the 192 STROBE entries, 139 (72.4%) entries were scores “Yes” for CoR, and 53 (27.6%) were scored “No” for CoR (**Figure 4**).

### Microbiome Methodological Consideration

**Figure 5:**
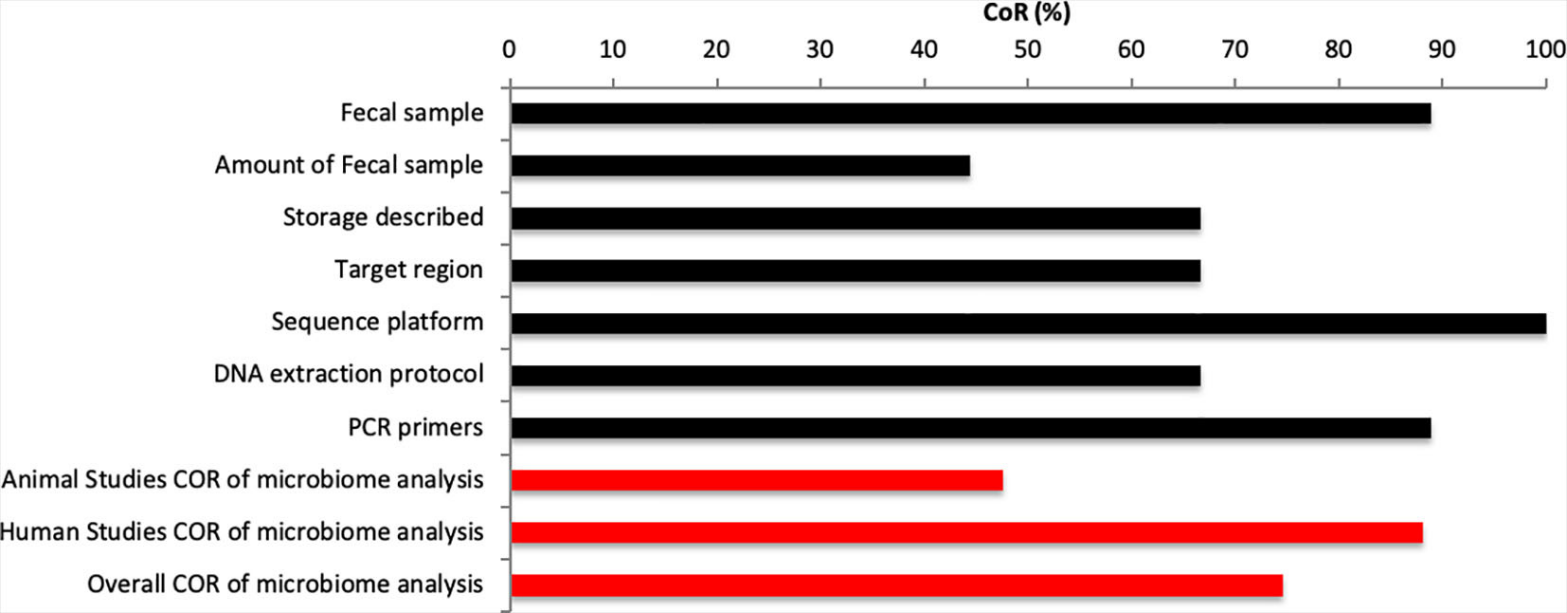
Microbiome methodological consideration.

The analysis included a total of 63 entries (7 entries per study). Of the 63 entries, 47 (74.6%) entries were scores “Yes” for CoR, and 16 (25.4%) were scored “No” for CoR (**Figure 5**). One study within this review did not use caecum samples rather than fecal samples (46). None of the animal studies reported the amount used for microbial analysis (46, 52, 53). Reporting for the amount of fecal sample varied within human studies with two studies failing to report amount used (40, 55). Similarly, mixed results were found for storage information given with two animal studies (52, 53) and one human study (55) not reporting the storage of samples between collection and analysis. Three studies did not report target regions (53–55). All studies reported the sequencing platform used (4, 40, 46, 52–57) and all human studies reported the DNA extraction protocol and PCR primers used (4, 40, 54–57). However, none of the animal studies reported the DNA extraction protocol (46, 52, 53) and one animal study failed to report PCR primers used. The major of publications within this review used 16S rRNA sequencing (4, 40, 46, 52, 53, 56, 57). Two studies used alternative techniques such as RT-qPCR for 16 s primers (54) or qPCR for 16 s primers (55). Studies, which reported target region investigated V3 or V4 regions (4, 46, 52, 56, 57). The preferred sequencing platform was the Illumina Miseq platform (4, 46, 52, 54, 56, 57).

Overall, we found a CoR score of 74.6% for all studies, which a large divide between CoR of animal (CoR: 47.6%) and human studies (CoR: 88.1%).

### Microbiome Analysis and Its Relationship to Behavior

In the following sections, we summarize the taxonomic changes in validated animal models of schizophrenia (46, 52, 53) and high-risk and UHR individuals (4), first-episode psychosis (54), first-episode schizophrenia (55), and individuals with chronic schizophrenia (40, 56, 57), all compared to healthy controls. The reviewed publications consistently report OTU (operational taxonomic unit) values, alpha and beta diversity, terms not frequently used outside the field of microbiome research. OTU is used to cluster sequences based on their similarities (60). Alpha diversity (within-sample) is the species number (richness) and distribution (evenness) within a host organism or habitat, showing “how many different species were found,” i.e., how many different bacteria are in a healthy individual, which can be measured using Shannon diversity index and Faith’s Phylogenetic Diversity (56). Beta diversity (between-samples) answers the question “How different is the microbial composition in one environment compared to another?”, calculated using Bray-Curtis dissimilarity and unweighted UniFrac and ordinated using principal coordinate analysis (PCoA).

**Figure 6:**
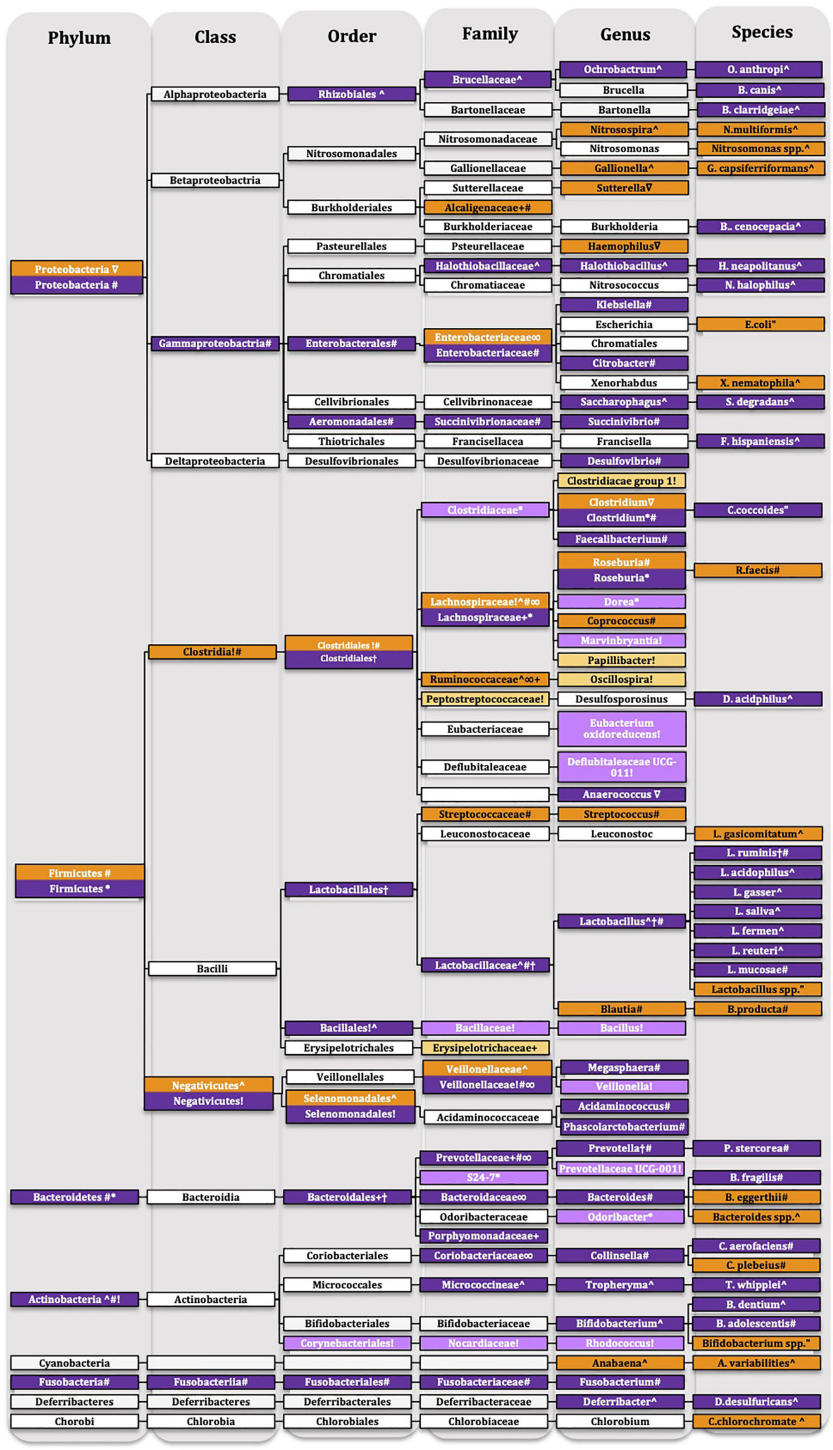
Taxonomic tree of schizophrenia. Showing reduced abundance in orange, increased abundance in purple with lighter shades of orange and purple to signify that only preclinical evidence is available. White-no change only for representative purposes. Ultrahigh-risk individuals †(4), first episode psychosis ^ (54), first episode schizophrenia “(55), chronic schizophrenia # (57) ∇(56) ∞(40), maternal immune activation model + (53), pharmacological model * (52), social isolation! (46).

Within the *Results* section we will be reporting changes according to phylum levels, this structure will remain for the discussion, however we will be discussion changes at lower taxonomic units within the phylum sections of the discussion. Details of the taxonomic changes are described in **Tables 1** and **2** and **Figure 6** showing reduced abundance in orange, increased abundance in purple with lighter shades of orange and purple to signify that only preclinical evidence is available.

#### Animals

One study investigated the gut microbiota of C57BL/6N offspring in the neurodevelopmental MIA model (53). MIA mice, from as early as three weeks, showed increased intestinal permeability, which was shown through increased translocation of fluorescein isothiocyanate-dextran across the intestinal epithelium (53). Alpha diversity is the species richness within a host organism or habitat, showing “how many different species were found,” i.e., how many different bacteria are in a healthy individual. This remains unaltered in MIA mice. Beta diversity, which reflects the species diversity to contribute to species evenness between microbial communities, i.e., how different was the diversity of bacteria between healthy controls compared to diseased individuals, was significantly altered by the MIA. PCoA (index of beta-diversity) showed that MIA samples clustered significantly differently to control samples, indicating different gut microbiome composition compared to control animals. The primary drivers of the gut microbiome changes concerning diversity were the classes *Clostridia* and *Bacteroidia*. MIA significantly altered families in the phyla *Bacteroidetes*, *Firmicutes*, and *Proteobacteria* compared to controls (53) (**Figure 6** and **Table 1**).

Subchronic administration of phencyclidine for seven days, a pharmacological model of schizophrenia, significantly separated the microbiota population compared to controls (beta-diversity) (52). Locomotor activity was increased in phencyclidine treated animals with seven days and four weeks wash-out period compared to controls, which indicates a schizophrenia-like behavioral phenotype (52) (**Table 1**). Seven days after treatment with phencyclidine, no change was found in alpha-diversity. However, a weak but significant alteration was found in beta-diversity compared to controls using the PCoA-analysis, which indicates the separation of the microbial communities. Phencyclidine-treated animals showed increased abundance in genera belonging to the phyla *Firmicutes* and *Bacteroidetes* (52) (**Figure 6** and **Table 1**). Four weeks after phencyclidine treatment, no changes in alpha-diversity and beta-diversity were found between groups. However, the abundance of genera within the phyla *Firmicutes* and *Bacteroidetes* were significantly increased in phencyclidine-treated animals (52) (**Figure 6** and **Table 1**).

Social isolation resulted in hyperactivity, anxiety-like behavior, and impaired contextual learning and memory, as well as reduced IL-6 and IL-10 levels in the hippocampus (46). Although no significant changes for alpha-diversity and beta-diversity were found, socially isolated animals showed a trend toward a decrease in alpha diversity and a trend towards differential clustering of microbial communities (beta diversity) (46) (**Figure 6** and **Table 1**). Social isolation increased the abundance of *Actinobacteria* at phylum level. At class, order, family, and genus level social isolation altered the abundance to both directions of the phyla *Firmicutes*, *Actinobacteria*, and *Bacteroidetes* (46) (**Figure 6** and **Table 1**).

In summary, the preclinical studies using translationally valid models for schizophrenia show somewhat inconsistent findings with the decreased abundance of the phylum *Proteobacteria* emerging as a partially shared feature (53). At the same time, *Actinobacteria* (46) and *Bacteroidetes* (46, 52, 53) were increased, whereas bacteria within the phylum *Firmicutes* show altered expression toward both directions.

#### Human Studies

High-risk and UHR individuals who have a higher likelihood of developing psychosis in the future did not differ in microbial richness, alpha-diversity. However, beta-diversity was altered in high-risk and UHR individual’s analysis compared to controls. UHR individuals had increased bacterial abundance at order, genus, and species levels in the phyla *Firmicutes* and *Bacteroidetes* compared to the other groups (4) (**Figure 6** and **Table 2**). It is important to note that clinically, UHR individuals showed more severe symptoms and functional impairments on the Scale of Prodromal Symptoms for screening of schizophrenic symptoms and the Global Assessment of Function Scale, Modified Version, respectively, than the other two groups (4). Functional profile analysis using *Phylogenetic Investigation of Communities by Reconstruction of Unobserved State*s (PICRUSt), which is a bioinformatics software for metagenomic functional predictions, predicted that short-chain fatty acids (SCFA) related to pyruvate synthesis, acetyl-CoA synthesis, and fatty acid biosynthesis initiation pathways were increased in UHR individuals compared to high-risk and controls, however only the acetyl-CoA synthesis pathway was significantly predicted (4) (**Table 2**). This profile corresponds with altered glucose metabolites, which is particularly interesting in the context of energy metabolism abnormalities that have been recently identified in schizophrenia [27-30].

Patients with first-episode psychosis, who received a relatively short antipsychotic treatment (median length of 20 days), showed no difference in alpha diversity (54) (**Figure 6** and **Table 2**). Individuals with first-episode psychosis had enrichment in the phylum Actinobacteria. At class, order, family, genus and species levels the overall abundance of bacteria were altered in the phyla *Firmicutes, Actinobacteria*, *Proteobacteria*, “*Deferribacteres,” Euryarchaeota, Cyanobacteria*, and *Chlorobi* in patients with first-episode psychosis compared to healthy controls (54) (**Figure 6** and **Table 2**). Physically active patients had a reduced abundance of *Firmicutes* at family level compared to active, healthy controls.

At baseline, multiple bacteria were decreased in drug naïve first-episode schizophrenia at species level belonging to the phyla *Actinobacteria*, *Proteobacteria*, and *Firmicutes* compared to controls (55) (**Figure 6** and **Table 2**).

Individuals with chronic schizophrenia with over ten years of antipsychotic medication were investigated and compared to healthy controls (57). Gut microbiota samples did not differ in alpha diversity, microbial richness, and diversity, from healthy controls. However, they showed differential clustering of microbial communities of chronic schizophrenic patients compared to respective controls (beta diversity). Furthermore, healthy controls showed more similar bacterial communities, tighter clustering, than patients with schizophrenia (57) (**Figure 6** and **Table 2**). At phylum level, *Proteobacteria* and *Fusobacteria* were significantly increased, and *Firmicutes* were less abundant in schizophrenia patients compared to controls. At class, order, family, genus, and species levels bacteria belonging to the phyla *Proteobacteria*, *Fusobacteria*, *Firmicutes*, *Bacteroidetes*, *Actinobacteria*, and *Euryarchaeota* were altered in chronic schizophrenia patients (57) (**Figure 6** and **Table 2**). Furthermore, using PICRUSt analysis, functional pathways were identified to be altered in individuals with schizophrenia, such as pathways responsible for the synthesis of vitamin B6, fatty acid, starch, sucrose, tryptophan, cysteine, methionine, and linoleic acid metabolism and the degradation of some xenobiotics (57).

Similar to the aforementioned study (57), no changes in alpha diversity were found in chronic, medicated patients with schizophrenia and schizoaffective disorder (56). However, beta diversity showed a clear separation between the patient and control populations and showed a wider distribution of schizophrenia samples (56) (**Figure 6** and **Table 2**). At phylum level, *Proteobacteria* were decreased in patients with schizophrenia. At genus level, bacterial abundance was altered bidirectional in the phyla *Proteobacteria, Proteobacteria*, and *Firmicutes* (56) (**Figure 6** and **Table 2**).

Another recent study has gone beyond just determining the bacterial abundance in the microbiome in people with schizophrenia and evaluated if behavioral phenotypes could be transferred through fecal microbial transplant from patients with schizophrenia to germ-free mice (40). The gut microbiome of patients with schizophrenia had reduced alpha diversity compared to healthy controls, suggesting a lower within-sample diversity. Furthermore, beta-diversity was also significantly altered in schizophrenia patients (40) (**Figure 6** and **Table 2**). Bacterial abundance was altered at family level belonging to the phyla *Firmicutes*, *Bacteroidetes, Actinobacteria*, and *Proteobacteria* (40) (**Figure 6** and **Table 2**). Animals, which received elements of the gut microbiome from patients with schizophrenia *via* fecal microbial transplants, showed a behavioral phenotype that had some overlaps with schizophrenia-like behaviors, including locomotor hyperactivity, reduced anxiety, and decreases depression-like behavior, attributed to increased activity. However, no difference was found in cognitive behaviors and sociability. Mice, which received fecal matter from individuals with schizophrenia, showed an increased startle response at high-decibel tones; however, no difference was found in pre-pulse inhibition of startle, which has been used extensively as a translational behavioral biomarker of psychotic states. Investigators further verified that gut composition was altered through the fecal microbial transplant and found that the gut microbiome significantly differed compared to that of control mice. The most changed bacterial families were *Aerococcaceae* (Phylum: *Firmicutes*), and *Rikenellaceae* (Phylum: *Bacteroidetes)*, which was similar to changes found in patients with schizophrenia [58].

Overall, the studies highlighted in this review demonstrated differential changes for all major phyla, including *Proteobacteria*, *Firmicutes*, *Bacteroidetes*, *Actinobacteria*, and *Fusobacteria* in patients with schizophrenia compared to healthy controls (4, 40, 54–57).

## Discussion

### Diversity of the Gut Microbiome in Schizophrenia

The diversity of the gut microbiome is unique to each individual. The majority of publications reviewed here identified no change in alpha diversity across animal and human studies (4, 46, 52–54, 56, 57). However, one study demonstrated an overall decrease in alpha diversity in individuals with chronic schizophrenia (40). The majority of patients were medicated with antipsychotics, which may have resulted in reduced microbial community diversity (40), as treatment with an atypical antipsychotic reduced microbiome community diversity in patients with bipolar disorder (alpha diversity), which was more profound in females (61). On the contrary, individuals on antipsychotic medications had no change in microbial community diversity (57). Due to conflicting results, we can only speculate that antipsychotic treatment may have resulted in a decrease in alpha diversity. Therefore, future studies should investigate the potential impact of antipsychotic treatment on the gut microbiome in schizophrenia.

The gut microbiome of patients with psychosis and animal models of schizophrenia are separated from the microbiota of healthy control individuals and control animals (beta diversity) (4, 40, 52–54, 56, 57). This clearly demonstrates that the gut microbiome in psychosis differs from that of the healthy controls. However, as there is a lack of studies comparing the microbiome between psychosis and other psychiatric and chronic, noncommunicable disorders, this does not necessary mean that the microbiome profile identified in psychosis is diagnostically specific. Nevertheless, these results, together with data showing that high-risk and UHR individuals have an altered microbiome compared to that of healthy controls (4), raise the possibility of developing the gut microbiome profiling further to be used as part of a biomarker battery to identify individuals at risk for later development of psychosis.

The studies outlined in this systematic review revealed that the phyla *Proteobacteria* (57), *Firmicutes* (52, 57), *Bacteroidetes* (52, 57) *Fusobacteria* (57), and *Actinobacteria* (46, 54, 57) were altered in patients at risk to develop schizophrenia, with chronic schizophrenia or in animal models of the disease at all different taxonomic units (**Figure 6**). First, we will individually discuss each of these phyla, within each phyla section we are discussing lower taxonomic units, and consider the functional implications that might be associated with their change.

#### Proteobacteria

Altered abundance, in both directions, of Proteobacteria has been associated with obesity, inflammation, and altered gut permeability (62–65). *Proteobacteria* were increased (57), as well decreased (56) in patients with chronic schizophrenia (**Figure 6** and **Table 2**) in the gut microbiome, but not in the oropharynx microbiome (66), which suggests that the imbalance of *Proteobacteria* abundance are specific to the gut microbiome. While an increased abundance of *Proteobacteria* is associated with the neonatal period (67), gastric bypass surgery (62, 68), and disease states such as metabolic disorders (69) and intestinal inflammation (70), decreased abundance of *Proteobacteria* have been found in overweight individuals (62). One study identified no difference in body mass index (57), whereas, in another study, individuals with schizophrenia had a significantly higher body mass index compared to healthy controls (56) (**Table 2**). Weight may have been a contributing factor influencing the abundance of *Proteobacteria*. Possibly, the decrease in *Proteobacteria* is more representative of patients with psychosis, because they tend to be more overweight or obese compared to mentally healthy controls (71). Therefore, future studies need to include lifestyle factors, such as diet, as potential covariates in the data analysis. At family level, both increased (57) and decreased (40) abundance of *Enterobacteriaceae* have been found (**Figure 6** and **Table 2**). An increase in *Enterobacteriaceae* have been associated with obesity (63), inflammation (64) and a “leaky gut” (65); however, obesity could not have been the driver for the change of this bacterial family as individuals with schizophrenia in both studies were in the healthy weight range based on their body mass index (40, 57). Increased gut permeability has been suggested to be related to schizophrenia (72) and may have been a contributing factor for the increase in *Enterobacteriaceae* (57). Besides, patients with schizophrenia show higher levels of proinflammatory cytokines (73). It is unclear, however, if the increased abundance of *Enterobacteriaceae* (57) was driven by proinflammatory cytokines or increased gut permeability in schizophrenia.

#### Firmicutes

An increased abundance of *Firmicutes* has been associated with an unhealthy dietary pattern, such as western diet and obesity (5, 74). A higher abundance of *Firmicutes* was found in the oropharynx microbiome of patients with schizophrenia (66). Animals injected with phencyclidine, a pharmacological model of schizophrenia, showed an increase in *Firmicutes* in their gut microbiome (74). In contrast, *Firmicutes* were less abundant in patients with chronic schizophrenia (57) (**Figure 6** and **Table 2**). At class, order and family levels *Negativicutes*, *Selenomondales*, and *Veillonellaceae* were reduced in first-episode psychosis (54) but were increased in an animal model of social isolation (46). *Veillonellaceae* were also increased in chronic schizophrenia (40, 57) (**Figure 6** and **Table 2**). Patients with chronic schizophrenia showed both an increase and a decrease in the genus *Clostridium* (56) in different studies (**Figure 6** and **Table 2**). Interestingly, an increase in this genus is associated with risperidone treatment (75). At this point, we are unable to conclude about the potential role of risperidone treatment as both studies (56, 57) report antipsychotic use, without specifying the type of medication used. Several species of *Clostridium* are precursors of 4-ethylphenylsulfate (76), which may contribute to the pathophysiology of schizophrenia, as it is important in pheromonal communication in mice under the control of testosterone levels (77). Serum 4-ethylphenylsulfate was elevated in MIA animals, a neurodevelopmental model of schizophrenia and 4-ethylphenylsulfate induced an anxiety-like behavioral phenotype, which may suggest behavioral abnormalities may be related to 4-ethylphenylsulfate produced by *Clostridium* (53). At species level, first-episode psychosis (54) and chronic schizophrenia patients (57), show similar to a study in oropharyngeal samples of schizophrenia patients, a significantly increased abundance of *Lactobacillus phage phiadh* (78) (**Figure 6** and **Table 2**).

#### Bacteroidetes

*Bacteroidetes* were increased in the pharmacological model of schizophrenia (52) and patients with chronic schizophrenia (57) (**Figure 6** and **Table 2**). Stress is involved in the pathophysiology of psychotic disorders, such as schizophrenia (79). In a mouse model of stress, mice were subjected to an aggressive male mouse within their home cage to induce stress. Investigators found elevated levels of the genus *Bacteroides* (80), which was demonstrated in chronic schizophrenia (57) (**Figure 6** and **Table 2**). Treatment with the probiotic *Bacteroides fragilis* was shown to improve anxiety-like, repetitive and communicative behaviors, and sensorimotor gating in the MIA model (53) (**Table 2**). *Bacteroides fragilis* improved anxiety-like (57), repetitive (57) and communicative behaviors (57), sensorimotor gating (57); however, *Bacteroides fragilis* do not influence social behaviors (53) (**Figure 6** and **Table 2**). Depressive-like symptoms in chronic schizophrenia patients were associated with an increased abundance of *Bacteroides* (56).

#### Actinobacteria

Actinobacteria are increased in first-episode psychosis, chronic schizophrenia, and socially isolated animals (46, 54, 57) (**Figure 6** and **Table 2**). *Tropheryma*, which belong to the phyla *Actinobacteria*, most studied species is *Tropheryma whipplei*, has been associated with intestinal malabsorption (81). *Tropheryma whipplei* was significantly increased in patients with first-episode psychosis (54) (**Figure 6** and **Table 2**). At genus level, *Collinsella* was elevated, which produces proinflammatory cytokines such as interleukin-17a and altered intestinal permeability in arthritis (82). Patients with schizophrenia show alter gut permeability (83). Additionally, multiple studies have found increases IL-17a plasma concentrations in naïve first-episode schizophrenia patients (84, 85).

In conclusion, the published literature indicates that schizophrenia, both first-episode and chronic, is associated with microbiota changes, as shown by beta diversity, that will distinguish them from healthy controls. In this systematic review we included studies in which healthy controls were compared to either individuals at risk to develop psychosis or with diagnosed psychosis (either first episode or chronic). Such cross-sectional design, together with a variety of cofounding factors, which were controlled for in some, but not in all cases, precludes us from concluding about causality. However, our review of the available human literature clearly indicates the existence of an association between different stages of psychosis and the gut microbiome. Future studies will be required to identify the primary drivers of the microbiome alterations in psychosis and the potential direction of causality between gut microbiome changes in psychosis.

### Methodological Considerations

#### Cofounding Factors

Within the reviewed publications gender was not addressed as a potential cofounding varible, which can potentially affect the gut microbiome. A review investigated gender differences in the gut microbiome and concluded that gender effects are inconsistent and identified that differences in geography, life style, diet, age, genetics, and potential other factors contribute more extensively to alterations in the gut microbiome (86). Within the reviewed articles, we reviewed populations from different geographical locations, which may have contributed to observed differences in microbiome. However, all studies demonstrated a clear separation between matched controls and psychotic individuals controlling for geographical location. Another major cofounding variable is age. We observed a broad age range in the reviewed studies, which may be expected considering that we included studies reporting on at-risk, UHR as well as on chronic schizophrenia. It has been suggested that age might be a major contributor to alterations in the gut microbiome (87). Gut microbiome changes within the elderly are associated with physiological changes within the gastrointestinal tract and has been demonstrated to reduce over time in diversity, shifts in dominant species, a decline in beneficial bacteria and decreased availability of beneficial metabolites such as short chain fatty acids (88). Older individuals have lower levels of Firmicutes and increased abundance of Proteobacteria (88). It is difficult to establish the potential cofounding effect of age on the results presented in the review. It should be noted, however, that each study contained age-matched controls, just like in the case of geographical location.

#### Fecal Sample Methodology Cofounders

We demonstrated that the CoR was greater in human studies allowing for greater comparability between studies. However, animal studies lacked reporting and should therefore be interpretate carefully. Overall, alterations and lack of reporting can be potential cofounding factors [further extensive review elsewhere (42)]. Methodological difference should be standardised in the future to improve translatability between animal and human studies and would allow for improved interpretation of data.

### Factors Potentially Contributing to the Altered Microbiome in Psychosis

In the following section we address potential factors influencing the gut microbiome in individuals with psychosis. Due to the heterogeneous nature of psychotic disorders no specific and unique factors to leading to psychosis are known. Therefore, we considered common life-style and environmental factors, genetic susceptibility and medication use that can potentially influence the gut microbiome in psychosis, as well as in other serious mental illness.

#### Environmental Factors

##### Early Life Events

Children will receive the first inoculum from their mothers (89). Mode of delivery can influence the gut microbiome (37). Offspring will receive during vaginal birth microbes found in the maternal vagina and feces (89). Whereas, during Cesarean delivery (C-section), most microbes colonizing the gut are from external body surfaces (89). C-section significantly decreases *Bifidobacteria* spp. (90) and increases *Staphylococcus*, *Streptococcus* or *Propionibacteria* (91, 92). However, the differences in microbiota between C-section and vaginal birth gradually disappear over time (37). Preterm birth will result in an increased abundance of *Proteobacteria* and a lack of *Bifidobacterium* and *Lactobacillus* at genus level (37). It has been established that early life events are potential risk factors for schizophrenia (93, 94). C-section and preterm birth have been linked as a risk factor for schizophrenia (93, 94). *Bifidobacterium* spp., which is in line with the microbial profile of C-section and preterm birth, were decreased in first-episode psychosis patients (55) (**Figure 6** and **Table 2**). Chronic schizophrenia patients had increased levels of *Proteobacteria* at phylum level, which is related to preterm birth (57) (**Figure 6** and **Table 2**). In first-episode psychosis, UHR and chronic patients with schizophrenia *Lactobacillus*, which is again related to preterm birth, was increased (4, 54, 57) (**Figure 6** and **Table 2**).

These early life events seem to alter the gut microbiome and may influence the development of psychosis later in life, perhaps through brain development influenced by the microbiome. Future research should specifically investigate if the changes in the microbiome due to the aforementioned factors contribute to the development of psychosis.

##### Stress

It has long been established that stress and the activity of the hypothalamus-pituitary-adrenal (HPA) axis can alter the composition of the gut microbiome (39). Maternal separation, a model of stress, results in prolonged HPA activity, which resulted in rhesus monkeys (95), rats (96) in altered microbiome composition (95). Chronic restraint stress in adult mice resulted in differential gut microbiota composition compared to nonstressed mice (80). Stress decreased *Bacteroides* spp. and increased *Clostridium* spp., which was accompanied by an activation of the immune system and a “leaky” gut (97), allowing for the translocation of luminal content such as lipopolysaccharides.

Stress is involved in the development of psychotic disorders such as schizophrenia (79, 98). Life events perceived as stressful can increase the occurrence of psychotic episodes (99). Individuals with schizophrenia experience stress more intensely; therefore even minor everyday stressors might exacerbate positive symptoms (100, 101). Management of day-to-day stress can be used in the management of psychosis (102); however, this intervention, in combination with antipsychotics, is only partially protective (103). This hypersensitivity to stress might be attributed to inappropriate autonomic nervous system and HPA axis function (79). Psychosis itself is a stressful event for the body, activating the stress response (98). Hypercortisolemia has been shown in patients with schizophrenia (104), which has been linked to the negative symptoms of schizophrenia (79). However, increased cortisol levels are not consistently found in individuals with schizophrenia (105). A meta-analysis found dysregulation of cortisol in psychotic patients (106). Allostatic load is the adaptation in response to stimuli such as stress (98). An increased allostatic load was seen in first-episode psychosis and schizophrenia patients compared to controls (98). This study found a positive correlation between positive symptoms with allostatic load in schizophrenia patients (98).

The genus *Clostridium* was increased in animals treated with phencyclidine (52) and chronic schizophrenia patients (57); however, Clostridium also decreased in chronic schizophrenia patients (56) (**Figure 6** and **Table 2**), consistent with a stress response. However, the stress level of schizophrenia patients was not described, and therefore it is unknown if the microbiota was altered due to increased stress or visa versa (57). A decrease in *Bacteroides* spp. is associated with stress (97), while in chronic schizophrenic patients, this genus was decreased (57) (**Figure 6** and **Table 2**).

In conclusion, based on the limited data available, it is difficult to establish if stress altered the microbiome. Future studies should assess stress levels and allostatic load to understand the impact of stress in psychosis on the gut microbiome.

##### Infectious Agents

Infectious agents, such as *Toxoplasma gondii*, have been suggested to contribute to the development of schizophrenia (107, 108). It has been recently demonstrated in a preclinical study that chronic *Toxoplasma gondii* infection results in an enrichment of Bacteroidetes in CD1 mice compared to noninfected controls (109). Interestingly, Bacteroidetes were also increased in a pharmacological model of schizophrenia (52) and in individuals with chronic schizophrenia (57). However, on the basis of the available evidence it is not possible to conclude about causality of the link between *Toxoplasma gondii* infection, alteration in the gut microbiome and the development of schizophrenia.

#### Lifestyle Factors

##### Diet

Diet is shaping the composition of the gut microbiome (5). The gut microbiome, in turn, is important in metabolizing the ingredients of food (18) and host fat storage, through the absorption of monosaccharides by the gut microbiota from the lumen of the gut, promoting hepatic lipogenesis by fasting-induced adipocyte factor suppression (110). Multiple studies have demonstrated that altering diet rapidly changes the gut microbiome (5, 111) and microbial beta-diversity (112). Early life nutrition, through changing the gut microbiome, is important in the infant’s development (113). Different diets in adulthood have been shown to modulate the gut microbiome, such as a high-protein, reduced carbohydrate diet (114), ketogenic diet (115), high fat, high sugar diet (western diet) (116, 117), and mediterranean diet (118). This indicates the strong impact of diet on the gut microbiome.

Nutrition is an important factor in schizophrenia due to poor dietary choices, causing obesity and secondary diseases (119). Obesity is twice as likely in schizophrenia/psychosis compared to the general population, affecting more than 50% of schizophrenic individuals (120). Drug naïve patients with schizophrenia show higher rates of obesity and type II diabetes compared to healthy individuals (120). Patients with schizophrenia consumed more fat (121, 122), saturated fat (122, 123), proteins (122), carbohydrates (122) and less fiber (121), than healthy controls. However, it was found that similar choices were made, but the overall food consumption was increased compared to healthy controls (122). Overall, people with psychosis tend to prefer unhealthy, fast food-type foods (120). This results in a dietary pattern high in saturated fats and sugars (“Western” diet). However, the gut microbiota of schizophrenia patients did not reflect that of individuals on a Western diet, characterized by a decrease in *Bifidobacteria*, *Bacteroides*, and *Prevotella* and an increase in *Firmicutes* (5). Within this review, first-episode psychosis had an increased abundance in *Bifidobacteria* (54) (**Figure 6** and **Table 2**). Different food types were not significantly associated with clustering of the microbiota (54). Increased *Prevotella* were found in UHR individuals (4); however, no conclusion can be drawn on the impact of nutrition as no metabolic or nutritional assessments were reported. In chronic schizophrenia patients, *Firmicutes* decreased, and the abundance of *Bacteroides* and *Prevotella* increased (57). It has to be noted that individuals in that study were Chinese patients, who did not show metabolic symptoms commonly seen in schizophrenia. This lack of weight gain has previously been reported in other Chinese schizophrenia patients (124). One study predicted an altered glucose metabolism (4), which is particularly interesting in the context of energy metabolism abnormalities that have been recently identified in schizophrenia [27-30]. A review investigated the link between the gut microbiome and glucose metabolism and found that the gut microbiome has substantial influence on glucose homeostasis through short chain fatty acids, bile acid metabolism, hormone secretion and synthesis of amino acids (125). However, future studies should address this point in individuals with psychosis to better understand the relationship between the gut, brain and energy metabolism.

In summary, individuals with psychosis show unhealthy dietary choices, which may influence the gut microbiome. However, the microbiome profiles described in studies on patients with psychosis do not support this notion. Nevertheless, at this point we can neither prove nor disprove the influence of diet to influence the gut microbiome in psychosis due to conflicting evidence and lack of reporting of dietary habits in these studies. Future studies should incorporate dietary patterns to be able to make a more definitive conclusion on the effects of dietary factors on the gut microbiome in individuals with psychosis.

##### Exercise

Exercise can impact microbial abundance in animals (126) and the human gut (127–131). The gut microbiota is different between sedentary individuals and people performing physical exercise (129, 132). Exercise reduced *Bacteroidetes* (129, 130) and *Proteobacteria* (132). Activity increased the undefined genus in the *S24-7* family (129), *Verrucomicrobia* (132), *Bifidobacteriaceae* (132), the *Streptococcaceae* family (129) and *Firmicutes* (130), compared to sedentary controls. In sedentary woman bacteria belonging to the families, *Barnesiellaceae* and *Odoribacteraceae* were more abundant compared to active women (130).

Individuals with schizophrenia and other psychotic disorders are significantly less physically active than healthy individuals, and are also less active then patients with other psychotic disorder (133, 134). Patients with chronic schizophrenia show an increased abundance in *Bacteroidetes* and *Proteobacteria* and a decreased abundance in *Streptococcaceae* and *Firmicutes* (57) (**Figure 6** and **Table 2**), which could possibly be mitigated by exercise as active individuals have opposing abundances (129, 130, 132). Further supporting this argument, *Firmicutes* were increased in phencyclidine treated animals (52), which could have been related to hyperactivity induced by the phencyclidine administration. The increase in the phylum *Bacteroidetes* is associated with a sedentary lifestyle (130). *Bacteroidetes* were increased in chronic schizophrenia patients (57) (**Figure 6** and **Table 2**); however, this increase was as well seen in the phencyclidine, hyperactive animals (52) (**Figure 6** and **Table 2**). Therefore, it is unclear at this stage if physical activity altered the gut microbiome. The activities of patients with schizophrenia are not explicit in the publications reviewed here. Nevertheless, physical activity has been considered to influence the gut microbiome composition in the context of schizophrenia Schwarz, Maukonen (54). Pateints with first-episode psychosis, stratified for amount of exercise, demonstrated an increased abundance of *Lactobacillaceae* and decreased abundance of *Veillonellaceae* at family level in physically active, first-episode psychosis individuals compared to physically active, healthy controls (54) (**Figure 6** and **Table 2**). Future studies need to assess physical activity levels as a potential cofounder to influence the gut microbiome.

##### Smoking

Environmental contaminants, such as smoking, influence the gut microbiota (135). Furthermore, smoking can lead to DNA damage and epithelial cell methylation (136), resulting in altered gut function and possibly altered microbiota composition. Smoking increased within-participant diversity, *Dialister invisus*, and *Megaspaera micronuciformis* were more abundant in the upper gastrointestinal tract in current smokers compared to the ones who never smoked (135). In rats, cigarette smoke decreased *Bifidobacteria* and SCFA, such as propionic and butyric acid (137), and increased *Lachnospiraceae* spp. (138). Passive smoking increases *Clostridium* spp. and reduces *Firmicutes* and *Enterobacteriaceae* in animals (139). In humans smoking increased *Clostridium* (140), *Bacteroidetes*, and *Proteobacteria* (141) and decreased *Firmicutes* and *Actinobacteria* (141).

Smoking is more prevalent in individuals with schizophrenia than in healthy individuals (142).

As reviewed above, in first-episode psychosis, *Bifidobacteria* were increased (54) (**Figure 6** and **Table 2**), whereas smoking decreased this bacteria. However, this study did not specify smoking status (54). In human studies, *Lachnospiraceae* (57) and *Firmicutes* (57) were decreased (**Figure 6** and **Table 2**). Decreased *Firmicutes* are in line with smoking; however, in patients with chronic schizophrenia, tobacco usage was not different compared to healthy control (57). At genus level contradictory results were found for *Clostridium*, however as mentioned before an increase in *Clostridium*, which is associated with smoking, was seen both in an animal model of schizophrenia (52) and in patients with chronic schizophrenia (57), where no difference in tobacco usage was seen between patients and controls. On the contrary, patients with schizophrenia, who were significantly likelier to smoke, had decreased abundance of Clostridium (56) (**Figure 6** and **Table 2**). *Enterobacteriaceae* were increased in chronic schizophrenia patients (41) (**Figure 6** and **Table 2**), which would be in line with possible tobacco usage; however, smoking status was not reported within that study.

In conclusion, according to the studies reviewed here individuals with schizophrenia either did not smoke more than the general population, or tobacco usage was not reported, except Nguyen, Kosciolek (56) where contradictory results were found with regards to smoking and the gut microbiome. Considering animal models of psychosis that do show altered gut microbiome despite the lack of smoking exposure, one can argue that the change in the gut microbiome seen in individuals with psychosis is likely to be independent of smoking status. However, most studies did not report smoking status, which predicts a firm conclusion regarding the link between smoking status and altered gut microbiome at this stage. Future studies should report smoking status and investigate if tobacco usage might be a cofounding factor influencing the gut microbiome.

Overall, although it has been widely acknowledged that life style-factors are essential in shaping the gut microbiome, the studies covered in this systematic review do not support the notion that the difference in the gut microbiome between controls and individuals with psychosis is causally related to lifestyle factors. Potentially, lifestyle factors, such as diet, exercise, and smoking may improve the gut microbiota of individuals with psychosis. Therefore, the completeness of reporting to provide a detailed account of the lifestyle factors is of great importance for future studies.

#### Genetics

Host genetic variation can influence the diversity of the gut microbiome (143). However, the relationship between host genetics and gut microbiota is largely unknown (143). Although, lifestyle factors such as diet and exercise will contribute to similar gut microbiota composition of close relatives, suggesting that genetics might be an important factor (143). For example, monozygotic twins share a more similar gut microbiota profile than dizygotic twins (143).

Genetic factors are important in the etiology of psychotic disorders such as schizophrenia (144). At this stage, no studies have investigated the effect of host genetic variation in individuals with schizophrenia on the gut microbiota. Therefore, we can only speculate that variations of bacterial abundances found in this population may be due to genetic variation involved in the pathogenesis of psychosis. Future studies should incorporate genetic analysis to understand the importance of host genetic variation on the gut microbiome.

#### Antipsychotics

Antipsychotics are the primary medications used for the management of schizophrenia (145). However, the knowledge of the effects of antipsychotics on the gut microbiome is currently in its infancy. Antipsychotic use can cause severe metabolic side effects such as weight gain, increased visceral fat, and glucose dysregulation (146), of which the mechanism of action is not fully understood (146). It is believed that the convergence of central and peripheral mechanisms are involved in metabolic side effects (146). It has been demonstrated that the composition of the gut microbiome is linked to obesity (147). Olanzapine treatment increases body weight (146, 148) and leads to a shift of the gut microbiome, which involves the increase of the phylum *Firmicutes* (146, 148) and decreases in the phyla *Bacteriodetes* (146, 148) in rodents. In female rats, olanzapine reduced the abundance of *Actinobacteria* and *Proteobacteria* compared to controls (148). Another study found an increase in *Erysipelotrichia* and *Gammaproteobacteria* and a reduced abundance of *Bacteroidia* at class level (149). Olanzapine inhibited the growth of *Escherichia coli* NC101 (149). One study found no change in microbial composition after olanzapine treatment, which may have been due to the short duration of treatment (150). However, this study demonstrated that acetate concentration changed, suggesting that olanzapine did affect on this by-product of the microbiome function (150). Risperidone increased *Firmicutes*, where *Lactobacillus* spp. were reduced, and decreased *Bacteroidetes*, where *Bacterioides* spp. were increased and *Alistipes* spp. decreased and *Proteobacteria* in female C57BL/6J mice compared to controls (151). The most abundant genera were *Allabaculum* spp. in risperidone treated animals compared to controls (151). Risperidone treatment resulted in weight gain (151). Transplant of fecal matter of female C57BL/6J mice on risperidone treatment to naïve mice resulted in a decreased resting metabolic rate, which may have contributed to the increase in body weight (151). Donor fecal matter was analyzed for risperidone concentration, which was 10-fold less than to establish a dose-response curve (151). Therefore, investigators concluded that the microbiota of risperidone treated animals was the obesogenic factor rather than the remaining risperidone within donor fecal samples (151). In medically healthy males, risperidone treatment led to weight gain with an altered gut microbiome compared to psychiatrically ill, but untreated patients. These findings with risperidone are similar to the gut microbiota changes after olanzapine administration. Antipsychotic treatment increased the abundance of *Lachnospiraceae* and decreased the abundance of *Akkermansia* after adjustment for body mass index in patients with bipolar disorder (61). However, *Akkermansia* was less abundant in nonobese, antipsychotic-treated patients (61). One publication covered in this systematic review assessed the changes in first-episode schizophrenia after 24-weeks of risperidone treatment (55). Chronic risperidone treatment altered metabolic parameters such as an increase in weight, body mass index, fasting serum glucose levels, triglycerides, and low-density lipoproteins (**Table 2**). Risperidone treatment increased *Bifidobacterium* spp. and *Escherichia coli* and decrease the abundance of *Clostridium coccoides* and *Lactobacillus* spp. (55) (**Figure 6** and **Table 2**).

#### Oxytocin

As the ever evolving literature recognizes the need for new drug treatments to complement the presently used antipsychotic medication, the neuropeptide oxytocin has been suggested as a potential novel treatment approach (152). Evidence from preclinical and clinical studies suggest therapeutic effects on all symptom domains of schizophrenia, with particular improvement in the negative symptoms (152). A potential link with the microbiome is suggested by the finding that the bacterium *Lactobacillus reuteri* upregulates oxytocin (153). One study we presented in this systematic review in first episode psychosis patients showed an increased abundance of *Lactobacillus reuteri* (54). However, the exact details of the interaction between oxytocin and the microbiome are currently unknown.

In our systematic review, six articles assessed different stages of the development of schizophrenia, such as high-risk, UHR, first-episode psychosis, first-episode schizophrenia, chronic schizophrenia. High-risk, UHR, and first-episode schizophrenia, first-episode psychosis patients were at study onset drug naïve (4, 54, 55). For chronic patients, antipsychotic treatment had to be more than six months of use. Therefore, changes might be cofounded by antipsychotic treatment (40, 56, 57). More studies are needed to identify the influence of antipsychotic medication on the gut microbiome.

### Functional Implication of the Change in Microbiota on the Psychopathology of Psychosis

#### Symptoms of Psychosis and the Gut Microbiome

Of the publications reviewed here, some have linked the gut microbiome to symptoms seen in schizophrenia. In the pharmacological model of schizophrenia, hyperactivity was associated with an increase in *Lachnospiraceae* and *Clostridiaceae* and at genus level an increase of *Roseburia, Clostridium*, and *Odoribacter* (52) (**Figure 6** and **Table 2**). In socially isolated animals, activity was positively correlated with the abundance of *Bacillales*. On the other hand, *Clostridales* was negatively correlated with locomotor activity (46). Socially isolated animals had increased locomotor activity in conjunction with reduced *Clostridales* (46). Furthermore, *Clostridiales* was negatively correlated with cognitive performance (46). Additionally, taxa belonging to the order *Clostridales*, at family level *Ruminococcaceae* and genus level *Papillibacter* were positively correlated to anxiety-like behaviors (46). *Bacillales* were negatively correlated to anxiety-like behaviors. Impaired contextual fear task, which investigates the associative learning process, was associated with an increase in *Veillonella* and *Defluvitaleaceae UCG-011* (46).

In people with schizophrenia, negative symptoms were related to decreased *Ruminococcaceae*, and self-reported mental well-being was positively correlated with the phylum *Verrucomicrobia* (56). Correlation analysis revealed that age of disease onset positively correlated with *Cyanobacteria* at phylum level, indicating that the earlier the disease onset, the higher the abundance of *Cyanobacteria*. Depressive-like symptoms were associated with an increased abundance of *Bacteroides*. *Veillonellaceae* OTU191 were negatively correlated with the Positive and Negative Syndrome Scale (40). On the other hand, *Bacteroidaceae* OTU172, *Streptococcaceae* OTU834, and two *Lachnospiraceae* OTUs (477 and 629) were positively correlated with PANSS (40). Bacterial numbers of Lactobacillus group, *Lachnospiraceae*, *Ruminococcaceae*, and *Bacteroides* spp. were correlated with symptom severity, particularly for negative symptoms and poorer functioning (54). Positive symptoms were correlated with bacteria of the *Lactobacillus group* (54).

Systemic biochemical changes and their relationship with the gut microbiome have been investigated. It was identified that *Blautia*, *Coprococcus*, and *Roseburia* were negatively associated with vitamin B6, taurine, and hypotaurine metabolic pathway and positively associated with methane metabolic pathways (57). Increased inflammatory cytokines have been seen in patients with schizophrenia (73). Hippocampal IL-10 and IL-6 correlated with *Peptostreptococcaceae* positively (46), while *Bacillales* correlated negatively with hippocampal IL-6 (46, 54). Interestingly, if the microbial composition of first-episode psychosis clustered with controls, these patients were more likely to show remission (70% of patients) (54). However, patients with abnormal microbial composition at baseline showed low remission rates (28% of patients) (54). Analysis revealed that this remission was not due to symptom severity at baseline (54). Clustering was not influenced by physical activity, body mass index, type of psychosis, duration of antipsychotic treatment, and the food consumed one week before fecal sample collection (54).

In summary, the abundance of specific bacteria correlated with behaviors and biochemical changes. This raises the possibility of targeted treatment approaches, such as pre/probiotics, to alleviate individual symptoms, however at this point in time causation has not been established. Further studies are required to link individual behaviors and biochemical changes in psychosis to the gut microbiome.

### Does the Microbiome Have a Pathogenic Role in Psychosis?

To address this question, we investigated retrospective studies to see if antibiotic use increased the prevalence of psychosis. A nationwide, register-based cohort study in Denmark outlined that antibiotics increased the risk of mental health disorders, which was independent of age, compared to antivirals and antimycotics (154). The risk for mental disorders increased in a dose-dependent manner, were a risk for mental disorders were more likely in individuals with more treated infections (154). The study showed that anti-infective agents were associated with an increased risk for schizophrenia spectrum disorders (154). Another study investigating the same Danish cohort identified that maternal infection during pregnancy treated with anti-infective agents increased the risk of mental disorders in the offspring, compared to offspring without maternal infection with anti-infective agents during pregnancy (155). The risk of mental health was increased if maternal infection treated with anti-infective medications occurred during the second and third trimester (155). It can be argued that maternal infection during pregnancy is known to be a risk factor to develop schizophrenia (156). However, Lydholm, Kohler-Forsberg (155) demonstrated that mental health disorder risk increased in response to maternal prescriptions during and after pregnancy. These recent studies suggest that antibiotic treatment during pregnancy and later in life may result in the later onset of schizophrenia. However, these studies are merely observational and would need further research to create causality.

Interestingly, animals, which received gut microbiome from patients with schizophrenia *via* fecal microbial transplant, showed a schizophrenia-like behavioral phenotype (40). Investigators further verified that gut composition was altered through the fecal microbial transplant and found that the gut microbiome significantly differed compared to that of control mice and was similar to that of patients with schizophrenia (40). Furthermore, the gut microbiota was altered in UHR individuals (4). Therefore, the changes in gut microbiota composition may contribute to the development of the disease pathophysiology. If confirmed, this raises the possibility to utilize the gut microbiome as an early biomarker for schizophrenia spectrum disorders.

Further research should be directed toward the understanding of bacteriophages, viruses that infect bacteria leading to the death of the bacteria or integration of the phage into the gut microbiome (78). Interestingly, a significantly increased abundance of *Lactobacillus phage phiadh* has been identified at species level in first-episode psychosis (54) and in patients with chronic schizophrenia (57), as well as in the oropharyngeal microbiome samples of patients with schizophrenia (78) (**Figure 6** and **Table 2**). However, the importance of bacteriophages is currently unknown and will require further investigation.

If alterations in the gut microbiome do indeed play a pathogenetic role in psychosis, agents, which modify the gut microbiome, can be considered as a potential modifiers of the disease process in schizophrenia. No research to date has investigated the effects of pre-or probiotics, antibiotics, fecal microbial transplant, or dietary intervention on the gut microbiome, specifically in people with schizophrenia. *Bacteroides fragilis*, a probiotic, in MIA model of schizophrenia in mice resulted in the restoration of gut permeability, gene expression, and IL-6 in the colon and normalized at family level *Lachnospiraceae* and *unclassified Bacteroidales* (53). Behaviorally, *Bacteroides fragilis* improved anxiety-like, sensorimotor, repetitive and communicative behavior; however, *Bacteroides fragilis* treatment did not affect sociability and social preference in MIA mice (53). Similar behavioral effects were found with *Bacteroides thetaiotaomicron*, whereas *Enterococcus faecalis* did not improve anxiety-like and repetitive behavior (53). Therefore, probiotic treatment might be a novel treatment for schizophrenia by improving gut functioning. However, a current review comes to the conclusion that the most recent evidence does not yet support the use of probiotics for the treatment of psychiatric disorders and more research is needed (157). Fecal microbial transplant, first performed approximately 1700 years ago (158), has not yet been described in either animal models or individuals with schizophrenia, but has been trialed for multiple sclerosis, patients showing normalization of neurological symptoms (159). These promising results may open up a new area in which the therapeutic effects of the microbiome can be taken advantage of. However, clinical trails are needed as currently only speculations can be made.

The antibiotic minocycline, which exerts neuroprotective and anti-inflammatory actions through supressing microglia activation and the modulation of excitatory neurotransmission, have attracted attention as potential treatments for schizophrenia, as shown by a number of small pilot studies with encouraging results (160). Although, a recent, large, randomized, placebo controlled clinical trial has provided unequivocal results showing no measurable therapeutic benefit on negative and other symptoms in patients with schizophrenia spectrum disorders (161), it has to be emphasized that active neuroinflammation involving microglial activation and neuropathology was unlikely to be present during the first years of schizophrenia, potentially explaining the lack of effect. The involvement of the gut microbiome in the mediation of the effects of minocycline has not been investigated.

Lastly, it is feasible to assume that diet could shift and normalize the gut microbiome in individuals with psychosis. The high fat, low carbohydrate ketogenic diet modifies the gut microbiome in an animal model of autism (115). Ketogenic diet increased *Enterobacteriaceae* and decreased *Firmicutes*, *Lactobacillus* spp., and *Roseburia* (115), which were altered in schizophrenia (**Figure 6** and **Table 2**). We have recently reported that ketogenic diet improves schizophrenia-like behaviors in an animal model of schizophrenia (162, 163), which can be due to the diet-induced alterations of the gut microbiome.

## Conclusion

Overall, the studies covered by this systematic review demonstrate that the gut microbiome in patients with schizophrenia spectrum disorders and animal models of schizophrenia is different from the microbiome of healthy controls. Once these initial findings are replicated and further extended in different patient populations the changes in the microbiome might be used as an independent biomarker of psychosis for high-risk and UHR individuals. While lifestyle factors do shape the gut microbiome, it is currently uncertain how they contribute to psychosis. Lifestyle changes such as diet, exercise, and cessation of smoking may influence the gut microbiome positively in individuals with schizophrenia. Stress and other early life events are possible further environmental factors contributing to this change. Multiple pathways by which alterations in the gut microbiome may have occurred, such as inflammation, the vagus nerve communication, stress response, and metabolites produced by the microbiota have been reviewed elsewhere (1, 5, 22). Clearly, more research is needed to clarify the role of specific host extrinsic and intrinsic factors and to identify specific mechanistic links, if any, between the gut microbiota and psychosis. Animal and human studies showed both similarities and differences in gut microbiota composition, which reflect the well-known difficulties to translate between preclinical and clinical research in the area of psychosis. Greater understanding and reporting of methodology of the gut microbiome will improve translatability from murine to human studies.

We conclude that the gut microbiome changes may precede the appearance of the diagnostic clinical symptoms of schizophrenia spectrum disorders and may contribute to the disease pathophysiology and the development of the behavioral symptoms. Further rigorous, well reported preclinical and longitudinal, mechanistically oriented clinical studies are needed to provide more evidence to support these potential links. Normalizing the altered gut microbiome with diet, pre- or probiotics, fecal microbiome transfer, or pharmacological interventions, may lead to improved symptom control and mitigation of the metabolic side effects of antipsychotic medication. However, randomized controlled clinical trials are urgently required to substantiate the potential use of targeting the microbiome as a novel therapeutic intervention in psychotic disorders.

## Data Availability Statement

The datasets used and/or analyzed during the current study are available from the corresponding author on reasonable request.

## Author Contributions

A-KK performed the data search, report selection, data extraction, and quality assessment and wrote the manuscript. RP was the independent assessor for the quality assessment and revised the final draft of the manuscript. ZS edited and revised all drafts of the manuscript.

## Funding

A James Cook University (JCU) Postgraduate Research Scholarship and a Higher Degree Research Enhancement Scheme from JCU supported A-KK and RP.

## Conflict of Interest

The authors declare that the research was conducted in the absence of any commercial or financial relationships that could be construed as a potential conflict of interest.

## Abbreviations

C-section, Cesarean delivery; CoR, completeness of reporting; GABA, gamma-aminobutyric acid; HPA, hypothalamic pituitary adrenal; MIA, maternal immune activation; NMDA, N-methyl-D-aspartate; OTU, operational taxonomic units; PCoA, principal coordinate analysis; PICRUSt, Phylogenetic Investigation of Communities by Reconstruction of Unobserved States; PRISM-P, Preferred Items for Reporting Systematic Reviews and Meta-analysis Protocols; RoB, risk of bias; SCFA, short-chain fatty acid; TNF, tumor necrosis factor.
